# Heteronemin, a Spongean Sesterterpene, Induces Cell Apoptosis and Autophagy in Human Renal Carcinoma Cells

**DOI:** 10.1155/2015/738241

**Published:** 2015-05-18

**Authors:** Szu-Ying Wu, Ping-Jyun Sung, Ya-Ling Chang, Shiow-Lin Pan, Che-Ming Teng

**Affiliations:** ^1^Pharmacological Institute, College of Medicine, National Taiwan University, No. 1 Jen-Ai Road, Section 1, Taipei 10051, Taiwan; ^2^Graduate Institute of Marine Biology, National Dong Hwa University, Pingtung 944, Taiwan; ^3^National Museum of Marine Biology and Aquarium, Pingtung 944, Taiwan; ^4^The Ph.D. Program for Cancer Biology and Drug Discovery, College of Medical Science and Technology, Taipei Medical University, No. 250 Wu-Hsing Street, Taipei 11031, Taiwan; ^5^Department of Pharmacology, College of Medicine, Taipei Medical University, No. 250 Wu-Hsing Street, Taipei 110311, Taiwan

## Abstract

Heteronemin is a bioactive marine sesterterpene isolated from the sponge *Hyrtios* sp. Previous reports have shown that heteronemin possesses anticancer activity. Here, heteronemin displayed cytotoxic effects against three human cancer cell lines (A549, ACHN, and A498) and exhibited potent activity in A498 human renal carcinoma cells, with an IC_50_ value of 1.57 *μ*M by MTT assay and a GI_50_ value of 0.77 *μ*M by SRB assay. Heteronemin initiates apoptotic cell death by downregulating Bcl-2 and Bcl-xL and upregulating Bax, leading to the disruption of the mitochondrial membrane potential and the release of cytochrome *c* from the mitochondria. These effects were associated with the activation of caspase-3/caspase-8/caspase-9, followed by PARP cleavage. Furthermore, heteronemin inhibited the phosphorylation of AKT signaling pathway and ERK and activated p38 and JNK. The specific inhibition of the p38 pathway by SB203580 or p38 siRNA treatment reversed the heteronemin-induced cytotoxicity and apoptotic signaling. Heteronemin also induced autophagy in A498 cells, and treatment with chloroquine (autophagy inhibitor) or SP600125 (JNK inhibitor) inhibited autophagy and increased heteronemin-induced cytotoxicity and apoptotic signaling. Taken together, this study proposes a novel treatment paradigm in which the combination of heteronemin and autophagy inhibitors leads to enhanced RCC cell apoptosis.

## 1. Introduction

Natural products are a source of compounds that sometimes have pharmacological activity that can be of therapeutic benefit in treating human diseases. Many compounds have potential anticancer effects involving multiple signaling pathways by mediating the complex signal transduction [1]. Recently, intense attention has been focused on marine natural products, such as pachymatismin, bryostatins, didemnin B, and bromovulone III [2–6]. Heteronemin, a marine sesterterpene isolated from the sponge* Hyrtios* sp., is endowed with an attractive pharmacological profile for drug development. Originally studied for its antimicrobial effects [7, 8], heteronemin has been reported recently as an apoptosis inducer, an inhibitor of tumor intravasation* in vitro* [9], and a potent modulator of the TNF*α*-induced NF-*κ*B pathway through the inhibition of the proteasome system [10].

Autophagy is an intracellular self-degradation process whereby double-membrane organelles termed autophagosomes deliver cytoplasmic materials to lysosomes [11]. The autophagosomes fuse with the lysosomes to become autolysosomes and the sequestered cargo is degraded [12]. Cells utilize the autophagy recycling system for removing both damaged cytosolic proteins and aged organelles to maintain quality and generate nutrient supply under adverse conditions. Besides these fundamental roles, autophagy is considered to be involved in the degradation of intracellular bacteria, antigen presentation, tumor suppression, cell survival, and cell death [12–15]. Whereas low autophagy levels promote cell survival, high autophagy levels cause catastrophic damage to a cell resulting in autophagic cell death [16]. Anticancer drugs induce autophagic and apoptotic cell death in various cancer cells [17, 18]. However, the interplay between autophagy and apoptosis is intricate. Autophagy can inhibit apoptosis by promoting cell survival, or autophagy and apoptosis may cooperate to induce cell death [19].

The members of the mitogen-activated protein kinase (MAPK) family are activated by cellular stress, UV light radiation, growth factor withdrawal, and proinflammatory cytokines, resulting in the regulation of cell proliferation, differentiation, survival, death, transformation, and adaptation [20–22]. The mammalian MAPK family comprises extracellular signal-regulated kinase (ERK), p38, and c-Jun NH2-terminal kinase (JNK, also known as stress-activated protein kinase or SAPK). In general, ERKs are associated with growth and proliferation, whereas JNKs and p38 are involved in cell death, including apoptosis [23]. p38 has four isoforms, p38*α*, p38*β*, p38*γ*, and p38*δ*. MKK3 and MKK6 phosphorylate p38 at Thr 180 and Tyr 182 and activate its kinase activity. In mammalian genomes, three genes encode the JNK family: JNK1, JNK2, and JNK3. The upstream MKK4 and MKK7 kinases mediate JNK activation via dual phosphorylation at Thr 183 and Tyr 185. The role of p38 and JNK in cell death is cell type- and stimuli-dependent. p38 can induce apoptosis through two different mechanisms: indirectly by promoting the transcription of proapoptotic genes or directly by activating Bax, Bim and belonging to the apoptosis-related Bcl-2 family proteins [24]. Furthermore, the phosphorylation of p38 acts as a mediator for caspase-8 in manganese-induced mitochondria-dependent cell death suggesting a strong link between p38 and the mitochondrial apoptotic pathway [25]. Conversely, the role of JNK in apoptosis is complex and it has been reported to have proapoptotic or antiapoptotic role or no role in the process. The role of JNK in autophagy is clearer: JNK signaling is required to upregulate LC3 in ceramide-induced autophagy in human nasopharyngeal carcinoma cells [26]. In addition, ROS is a strong activator of the JNK-AP-1 signaling pathway and plays an important role in JNK-dependent autophagy regulation [27, 28]. Analyzing the role of JNK in the crosstalk between apoptosis and autophagy remains an important challenge.

In this study, we report that heteronemin induces apoptosis and autophagy in the human renal cell carcinoma (RCC) A498 cell line. We demonstrate the role of p38 in heteronemin-induced cell apoptosis and the role of JNK in heteronemin-induced autophagy. We show in this system that inhibiting autophagy leads to a significantly enhanced cell death and apoptosis response. Heteronemin also inhibits the AKT signaling pathway and the phosphorylation of ERK. Our study is the first report that provides evidence that, besides apoptosis, heteronemin also induces autophagy in A498 cells. We propose that the combination of heteronemin with autophagy inhibitors leads to enhanced apoptosis in A498 cells.

## 2. Materials and Methods

### 2.1. Reagents and Chemicals

Heteronemin was extracted from marine sponge* Hyrtios erecta* and purified in Professor Ping-Jyun Sung's Lab. Minimum Essential Medium (MEM), RPMI 1640 medium, fetal bovine serum (FBS), penicillin, and streptomycin were obtained from Gibco BRL Life Technologies (Grand Island, NY). EGTA, EDTA, leupeptin, dithiothreitol, phenylmethylsulfonyl fluoride (PMSF), propidium iodide (PI), dimethyl sulfoxide (DMSO), MTT (3-[4,5]-2,5-diphenyltetrazolium bromide), 4′-6-diamidino-2-phenylindole (DAPI), SB203580, SP600125, and chloroquine were obtained from Sigma (St. Louis, MO). Antibodies to various proteins were obtained from the following sources: anti-mouse and anti-rabbit IgGs, poly-ADP-ribose polymerase (PARP), Bcl-2, Bcl-xL, Bax, and p62 antibodies were purchased from Santa Cruz Biotechnology (Santa Cruz, CA); p-AKT (Ser 473), AKT, p-ERK (Thr 202/Tyr 204), ERK, p-p70S6K (Thr 421/Ser 424), p70S6K, p-4EBP1 (Thr 37/46), 4EBP1, p-JNK (Thr 183/Tyr 185), JNK, p-p38 (Thr 180/Tyr 182), p38, p-HSP27 (Ser 78), Atg5, cleaved caspase-3, caspase-9, and caspase-8 were purchased from Cell Signaling Technology (Boston, MA); cytochrome *c* was purchased from BD Biosciences (San Diego, CA); caspase-3 was purchased from Imgenex (San Diego, CA); LC3 was purchased from Novus (Littleton, CO); actin and GAPDH were purchased from Millipore (Billerica, MA).

### 2.2. Cell Culture

Human cancer cell lines A549, ACHN, and A498 were purchased from the American Type Culture Collection (Manassas, VA). Cell lines were maintained in either RPMI 1640 medium (A549 and ACHN) or Minimum Essential Medium (A498) containing 10% heat-inactivated fetal bovine serum and 1% penicillin/streptomycin at 37°C under a humidified atmosphere with 5% CO_2_.

### 2.3. Cytotoxicity Assay

Cells were plated in 96-well plates for 24 h. The medium was removed, and the cells were treated with various concentrations of heteronemin. After treatment, 100 *μ*L MTT solution (0.5 mg/mL in phosphate-buffered saline (PBS)) was added to each well. After 1 h incubation at 37°C, MTT solution was removed and DMSO was added to dissolve dye. Absorbance at 550 nm was measured using a microplate reader (Thermo Multiskan GO, Waltham, MA), using RPMI or MEN medium as a blank.

### 2.4. Sulforhodamine B (SRB) Assay

Cells were seeded in 96-well plates in complete media. After overnight culture, cells were treated with various concentrations of heteronemin for 48 h and then cells were fixed with 10% trichloroacetic acid (TCA) and SRB at 0.4% (wt/vol) in 1% acetic acid. SRB bound cells were solubilized with 10 mM Trizma base and absorbance was read at a wavelength of 515 nm by using a microplate reader. Growth inhibition of 50% (GI_50_) is calculated as described previously [29].

### 2.5. *In Situ* Labeling of Apoptotic Cells

Heteronemin-induced A498 cell apoptosis was detected using the terminal deoxynucleotidyl transferase-mediated nick-end labeling (TUNEL) staining assay. Briefly, cells were seeded in 4-well chamber slides. After overnight culture, cells were exposed to 3 *μ*M heteronemin for 24 h and then fixed for 10 min by using ice-cold 1% paraformaldehyde. Staining was performed according to the TUNEL staining protocol provided by Promega Corporation (Madison, WI). Finally, photomicrographs of the TUNEL-stained cells were observed and photographed using Axioplan 2 fluorescence microscope (Carl Zeiss, Jena, Germany) equipped with a CCD camera (Nikon, Japan) at 20x magnification. Data were analysed by AxioVision software.

### 2.6. Cell Death Detection Assay

Cell death detection ELISA (Roche Applied Science, Indianapolis, IN) was used to quantify histone-complexed DNA fragments (nucleosomes) in cytoplasm of the apoptotic cells after induction of apoptosis. The manufacturer's protocol was applied from Roche and data were measured by microplate reader. Data were calculated and compared with those of a control group.

### 2.7. Western Blot Analysis

Whole cell lysates were prepared by extracting proteins using a lysis buffer. Proteins were size-fractionated by 10–12% SDS-PAGE and transferred electrophoretically onto polyvinylidene difluoride membranes. The membranes were sequentially hybridized with primary antibody and followed with a horseradish peroxidase-conjugated secondary antibody. Finally, the membranes were visualized using an enhanced chemiluminescence kit (VISUAL PROTEIN, Taiwan).

### 2.8. Measurement of the Change of Mitochondrial Membrane Potential (ΔΨ_**m**_)

Mitochondrial membrane potential was monitored by FACScan flow cytometric analysis. Cells were treated with heteronemin for the indicated time periods. Thirty minutes before the termination of incubation, the rhodamine 123 solution (final concentration of 5 mM) was added to the cells and incubated for the last 30 min at 37°C. The media were removed and cells were washed once with phosphate-buffered saline (PBS). After detachment by trypsinization, cells were resuspended in PBS and subjected to FACScan analysis.

### 2.9. Preparation of Cytosolic and Mitochondrial Fractions

Cytosolic fraction was isolated by using Cytochrome *c* Releasing Apoptosis Assay kit from BioVision Research Products (Mountain View, CA, USA). Briefly, after treatment, cells were harvested by trypsinization, washed once in ice-cold PBS, and resuspended in Cytosol Extraction Buffer. After incubation on ice for 10 min, cells were homogenized by gentle douncing (100 strokes) in a glass microgrinder and centrifuged at 700 g for 10 min at 4°C to pellet nuclei and unbroken cells. Supernatants from the centrifugation were further centrifuged at 10 000 g for 30 min at 4°C to get cytosolic fraction (supernatant) and mitochondrial fraction (pellet). The levels of cytochrome *c* in the cytosolic fractions were detected by western blot analysis.

### 2.10. Small Interfering RNA Transfection

Small interfering RNA (siRNA) against p38, Atg5, and the negative control was purchased from Ambion (Austin, TX), and the assay was performed as described previously [30]. Briefly, A498 cells were seeded in 6 cm dishes overnight and then transfected with 10 *μ*L negative control and siRNA (200  pmol) by using 10 *μ*L Lipofectamine 2000 (Invitrogen, Carlsbad, CA). After 24 h, the medium was replaced with growth medium and cells were treated with heteronemin for the indicated time.

### 2.11. GFP-LC3 Localization by Fluorescence Microscopy

Autophagy was confirmed by the presence of fluorescent puncta in cells transfected with a GFP-LC3. Briefly, A498 cells were seeded in 4-well chamber slides and transiently transfected with 2 *μ*g/mL GFP-LC3 plasmid (purchased from Addgene, Cambridge, MA) using Lipofectamine 2000. After 24 h, cells were treated with 3 *μ*M heteronemin for 6 h and then fixed with 4% paraformaldehyde and stained with DAPI for nuclei observation. The staining was examined and photographed using Axioplan 2 fluorescence microscope equipped with a CCD camera at 20x magnification. Data were analysed by AxioVision software.

### 2.12. Statistical Analysis

All experiments were performed at least three times. Data are expressed as mean ± SE for the indicated number of separate experiments. Statistical analysis of data was done with Student's *t*-test. *P* values less than 0.05 were considered significant.

## 3. Results

### 3.1. Heteronemin-Induced Cell Apoptosis in A498 Cells

**Figure 1:**
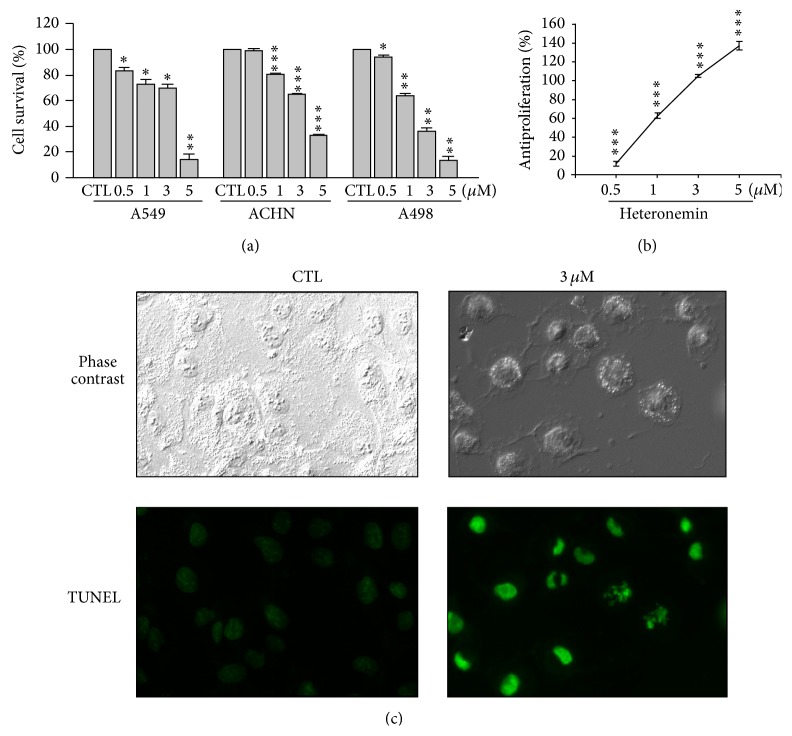
Effects of heteronemin on cell viability in human cancer cell lines. A549, ACHN, and A498 were treated with DMSO or heteronemin at various concentrations for 24 h for MTT assay (a) and for 48 h for SRB assay in A498 cells (b). Fluorescence microscopy of untreated or heteronemin-treated A498 cells for 24 h followed by TUNEL staining (at 20x magnification) (c). Data are expressed as the mean percentage of control ± S.D. of three independent experiments. ^∗^ *P* < 0.05, ^∗∗^ *P* < 0.01, and ^∗∗∗^ *P* < 0.001 compared with the control group. DMSO was used as the vehicle control (CTL).

We assessed the impact of heteronemin treatment on three human cancer cell lines: A549, ACHN, and A498. Using the MTT assay, we first measured cell viability. Heteronemin induces cytotoxicity in a concentration-dependent manner in the ACHN and A498 human renal carcinoma cell lines but not in the lung adenocarcinoma epithelial cell line A549. The cytotoxic activity against ACHN and A498 cell lines showed IC_50_ values of 3.54 *μ*M and 1.57 *μ*M, respectively (Figure 1(a)). With the IC_50_ values being in the same range, we chose to continue our investigation using only the A498 cell line.

We used the SRB assay to determine the antiproliferative activity of heteronemin in A498 cells. We treated the cells with different concentrations of heteronemin and found that heteronemin shows a potent antiproliferative effect with a GI_50_ value of 0.77 *μ*M (Figure 1(b)).

**Figure 2:**
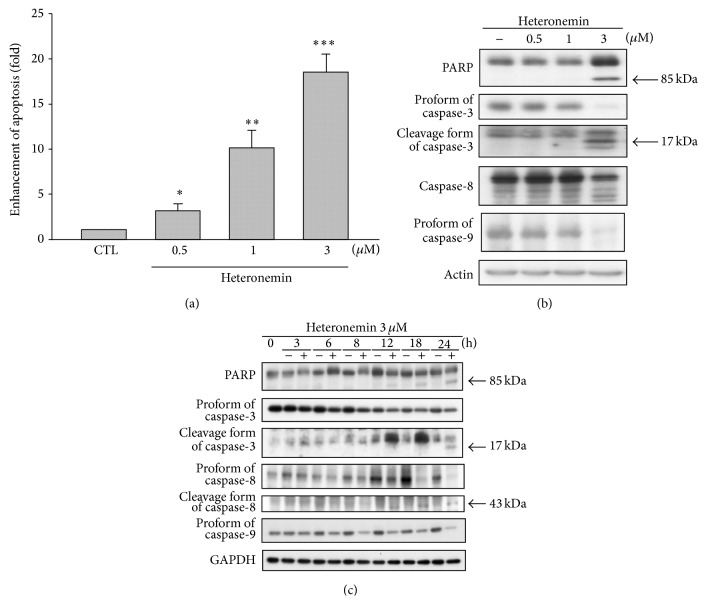
Effects of heteronemin treatment on cell apoptosis induction and expression of apoptosis-related proteins in A498 cells. (a) Cells were treated with DMSO or heteronemin at various concentrations (0.5, 1, and 3 *μ*M) for 24 h. Formation of cytoplasmic DNA was quantitatively measured by cell death ELISA^PLUS^ kit. Data are expressed as the mean percentage of control ± S.D. of three independent experiments. ^∗^ *P* < 0.05, ^∗∗^ *P* < 0.01, and ^∗∗∗^ *P* < 0.001 compared with the control group. A498 cells were incubated in the absence or presence of heteronemin at various concentrations (0.5, 1, and 3 *μ*M) for 24 h (b) or treated for indicated times (c), and cells were harvested and prepared for detection by western blotting. DMSO was used as the vehicle control (CTL).

We next investigated whether the reduced cell viability was due to apoptosis or necrosis using* in situ* labeling. Using TUNEL staining, heteronemin induced DNA fragmentation in A498 cells (Figure 1(c)). In light-field pictures, A498 cells appeared to be spindle-shaped, adhered to the surface of the culture plate, and were confluent after 24-hour incubation. After treatment with 3 *μ*M heteronemin, typical apoptotic features were observed such as cell rounding, cell shrinkage, and plasma membrane blebbing (Figure 1(c), upper panel). We also demonstrated that heteronemin increases the sub-G1 phase population in a concentration- and time-dependent manner (Figure S1 in the Supplementary Material available online at http://dx.doi.org/10.1155/2014/738241), which is consistent with a role in apoptotic cell death induction. Heteronemin triggered concentration-dependent apoptosis was independently confirmed using the cell death Detection ELISA kit (Figure 2(a)). In addition, heteronemin treatment resulted in the cleavage of PARP, caspase-3, and caspase-8 and the decrease of procaspase-9 in a concentration- and time-dependent manner (Figures 2(b) and 2(c)). Taken together, these results demonstrated that heteronemin induced cell death via the apoptotic pathway.

### 3.2. Heteronemin-Induced Apoptosis via a Mitochondrial-Mediated Pathway

**Figure 3:**
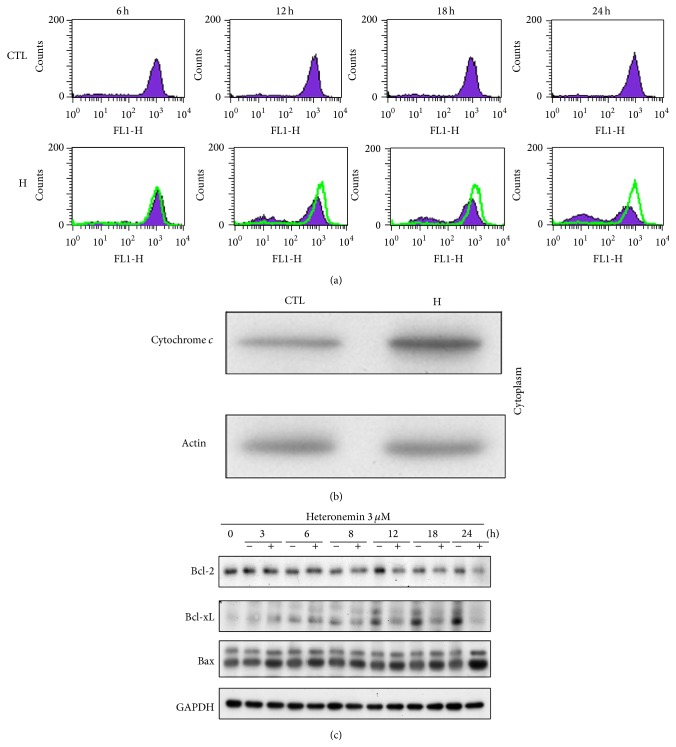
Effect of heteronemin on reduction of Δ*ψ*_*m*_ and release of cytochrome *c*. A498 cells were incubated in the DMSO or 3 *μ*M heteronemin for indicated time, and cells were harvested and prepared for detection, (a) mitochondria membrane potential by using FACScan analysis, (b) release of cytochrome *c* in cytosol for 24 h, and (c) Bcl-2, Bcl-xL, and Bax expression by using western blotting analysis. DMSO was used as the vehicle control (CTL).

The pivotal role of mitochondria in apoptosis induction is well established in mammals [31, 32]. In many types, cytochrome *c* is released from the mitochondrial intermembrane space in response to apoptotic stimuli. Cytochrome *c* is required for the assembly and the activity of the apoptosome, which is composed of the apoptosis-protease activating factor 1 (Apaf-1) and the initiator caspase, caspase-9 [33]. Heteronemin treatment caused loss of the mitochondrial membrane potential in a time-dependent manner and induced the release of cytochrome *c* into the cytosol (Figures 3(a) and 3(b)). In addition, the Bcl-2 protein family regulated mitochondrial permeability and the release of cytochrome *c* to control cell apoptosis. Heteronemin downregulated the expression of Bcl-2 and Bcl-xL and upregulated the expression of Bax (Figure 3(c)). These results suggested that heteronemin induced cell death in A498 cells through the mitochondrial apoptosis pathway.

### 3.3. Heteronemin Inhibited PI3K/AKT Pathway and ERK in A498 Cells

**Figure 4:**
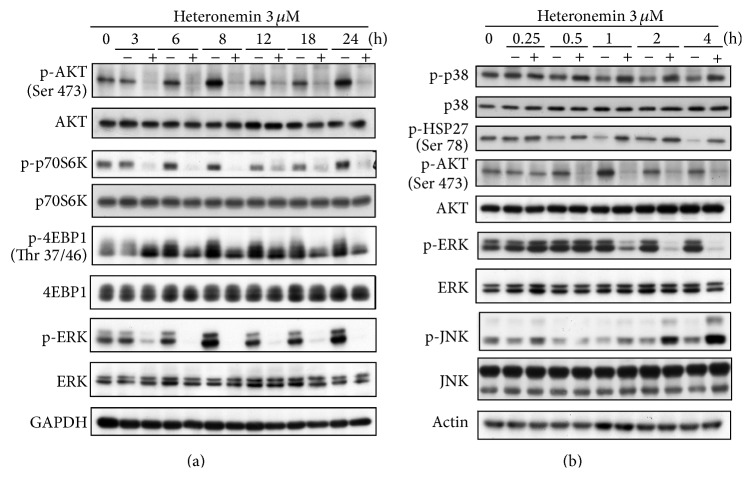
Effects of heteronemin on PI3K/AKT and MAPK signaling pathway in A498 cells. A498 cells were incubated with DMSO or 3 *μ*M heteronemin for the indicated time periods. After treatment, cells were harvested and lysed for detection of the expression of indicated protein via western blotting. DMSO was used as the vehicle control (CTL).

The PI3K/AKT and MAPK signaling pathways are major pathways regulating the apoptotic process. Thus, we determined whether heteronemin affects these pathways via the expression levels of their downstream signaling proteins. Heteronemin treatment significantly decreased the phosphorylation of AKT, p70S6K, 4EBP-1, and ERK in 3–24 h (Figure 4(a)). Our results also show that heteronemin treatment fast decreased the phosphorylation of ERK and AKT within 0.5–1 h (Figure 4(b)). In addition, we examined the phosphorylation of other MAPKs, JNK, and p38, previously linked to apoptosis as a response to stress [23, 34]. Western blotting revealed that heteronemin increased the phosphorylation of p38, p38 downstream effector HSP27, and JNK. In summary, our results demonstrated that AKT/p70S6K/4EBP1 signaling pathway and ERK are blocked by heteronemin, whereas p38 and JNK are activated by heteronemin in A498 cells.

### 3.4. p38 Played an Essential Role in Apoptosis Induction by Heteronemin

**Figure 5:**
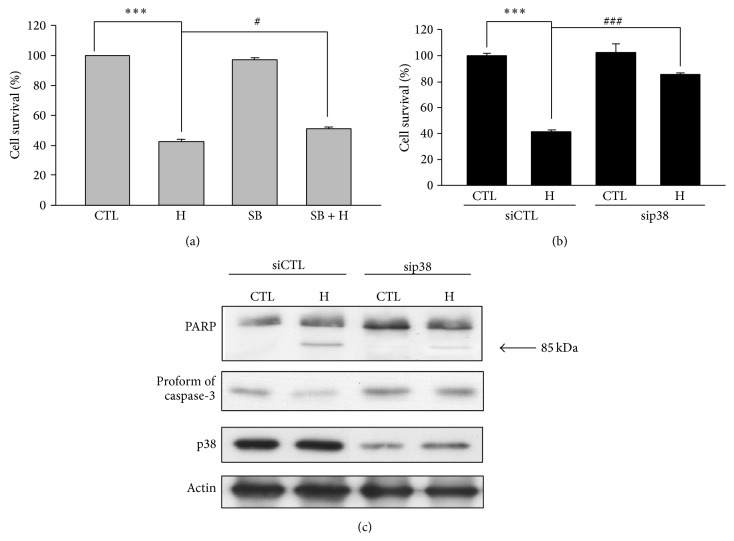
p38 involved in heteronemin-induced cell apoptosis in A498 cells. (a) p38 inhibitor, SB203580, was preincubated for 30 min and cell viability was determined by MTT assay. ^∗∗∗^ *P* < 0.001 compared with the control group. ^#^ *P* < 0.05 and ^###^ *P* < 0.001 compared with the heteronemin-treated group. p38 siRNA was transfected to evaluate the role in heteronemin-induced A498 cell death by using the MTT assay (b), expression of apoptosis-related proteins (PARP and procaspase-3) (c) for 24 h. H and SB are indicated as heteronemin 3 *μ*M and SB203580 25 *μ*M, respectively. CTL is indicated as control.

To further delineate the role of p38 in heteronemin-induced cytotoxicity, we used the p38 inhibitor SB203580. The results showed that SB203580 reversed the effect of heteronemin-mediated suppression of cell viability (Figure 5(a)). Moreover, transient transfection with p38 siRNA leads to the downregulation of p38 and significantly prevented heteronemin-induced cell death (Figure 5(b)), inhibiting the cleavage of PARP and the downregulation of procaspase-3 (Figure 5(c)). These results demonstrated that heteronemin-induced cell apoptosis is mediated through p38 regulation.

### 3.5. Heteronemin Induced Autophagy in A498 Cells

**Figure 6:**
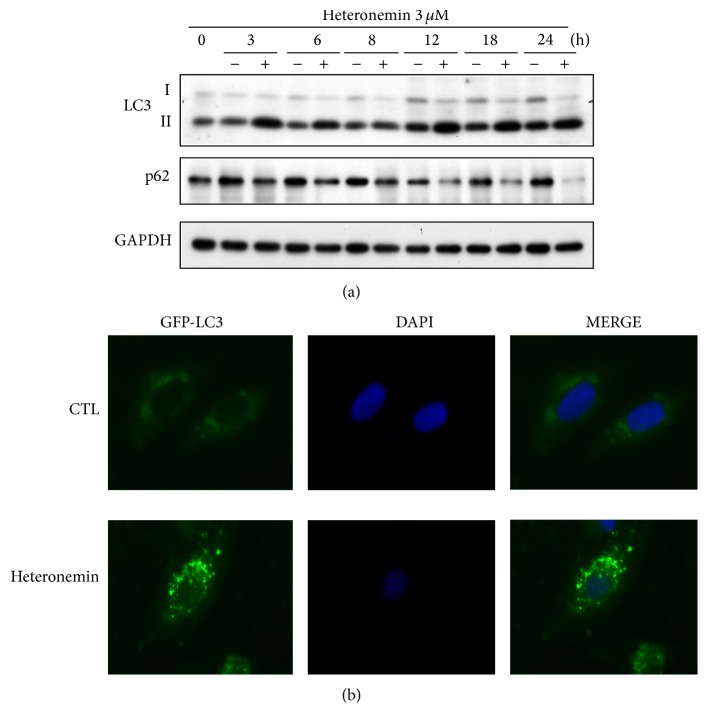
Effects of heteronemin on autophagy in A498 cells. (a) Cells were treated with 3 *μ*M heteronemin for indicated times, and cell lysates were subjected to western blot analysis of the expression of LC3 and p62. DMSO was used as the vehicle control (CTL). (b) Microscopic analysis of the effect of DMSO or heteronemin on the pattern of GFP-LC3 fluorescence. A498 cells were transfected with vectors encoding GFP-LC3, cultured in complete medium for 24 h, and treated for 18 h with DMSO or 3 *μ*M heteronemin. Representative images of GFP-LC3 puncta are shown, as photographed under microscopy.

The conversion of soluble LC3-I to lipid bound LC3-II via proteolytic cleavage and lipidation can be used as a marker for autophagy induction [35]. Western blotting revealed that heteronemin treatment markedly induced the expression of LC3-II in A498 cells, suggestive of autophagy induction (Figure 6(a)). During autophagy, p62 is wrapped into autophagosome and degraded in autolysosome. In heteronemin-treated cells, the expression of p62 was reduced as revealed by western blotting analysis, and this decrease correlates with the induction of autophagy. Furthermore, we assessed the ability of heteronemin to facilitate the conversion of LC3-I to LC3-II in GFP-LC3-expressing A498 cells. After heteronemin treatment, the GFP-LC3-transfected A498 cells showed a significant increase of punctate cytoplasmic dots, indicating autophagosome formation [36] (Figure 6(b)). Taken together, these data demonstrated for the first time that heteronemin induced autophagy in A498 cells.

### 3.6. Inhibition of Heteronemin-Induced Autophagy Enhanced Apoptosis in A498 Cells

**Figure 7:**
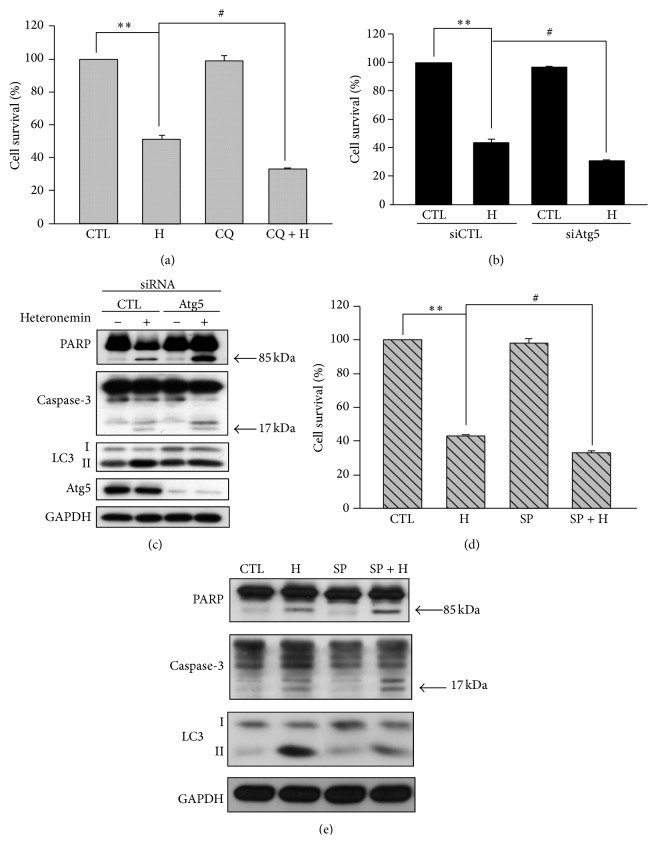
Inhibition of autophagy enhanced the anticancer effect of heteronemin in A498 cells. A498 cells were pretreated with autophagy inhibitor, chloroquine, for 30 min, then 3 *μ*M heteronemin was added for 24 h, and (a) the cell viability was determined using MTT assay. A498 cells were transfected with Atg5 siRNA or negative control and (b) the cell viability was determined using MTT assay and (c) the expression of apoptosis-related proteins (PARP and procaspase-3) and autophagy-related proteins (LC3 and Atg5) was evaluated for 24 h by western blotting. A498 cells were pretreated with JNK inhibitor, SP600125, for 30 min, then 3 *μ*M heteronemin was added for 24 h, and (d) the cell viability was determined using MTT assay and (e) the expression of apoptosis-related proteins (PARP and procaspase-3) and LC3 was evaluated for 24 h by western blotting. H, CQ, and SP are indicated as heteronemin 3 *μ*M, chloroquine 50 *μ*M, and SP600125 20 *μ*M, respectively. ^∗∗^ *P* < 0.01 compared with the control group. ^#^ *P* < 0.05 compared with the heteronemin-treated group. CTL is indicated as control. DMSO was used as the vehicle control (CTL).

The role of autophagy in cancer remains controversial. Autophagy can induce autophagic cell death through an overdegradation of the cytoplasm, or it can protect cancer cells from apoptosis by reducing the cellular stress [37]. To clarify the function of autophagy in heteronemin-induced cell death, chloroquine, an inhibitor of autophagy, was used. When cells were cotreated with chloroquine and heteronemin, heteronemin-induced cell death increased (Figure 7(a)). Because of Atg5 involved in the elongation of isolation membrane in autophagosome formation, we confirmed this effect by transfection A498 cells with Atg5-targeting siRNA (Figure 7(b)) and found that heteronemin promoted the cleavage of PARP and caspase-3 after Atg5 knockdown (Figure 7(c)).

Heteronemin induced the phosphorylation of JNK (Figure 4(b)). Using the JNK inhibitor SP600125 together with heteronemin, we showed (Figures 7(d) and 7(e)) that cytotoxicity and the cleavage of PARP and caspase-3 were intensified in the same way compared to when cells are cotreated with chloroquine or with Atg5-targeting siRNA. Taken together, these data suggested that when heteronemin-induced autophagy is inhibited, cytotoxicity and the apoptotic effects of the drug are potentiated.

## 4. Discussion

The ocean has a rich ecosystem and has recently attracted a lot of interest as a source of natural products for drug discovery. To date, approximately 16,000 marine natural products have been isolated from marine organisms and several of them exhibit a biological activity [38]. The ocean is now considered a source of potential drugs.

Many bioactive marine compounds are terpenes. Sesterterpenes and triterpenes are common terpenes extracted from sponges. Most bioactive compounds from sponges can be classified as anti-inflammatory, antitumor, immunosuppressive or neurosuppressive, antiviral, antimalarial, antibiotic, or antifouling [39]. Here, we investigated the anticancer activity of heteronemin, a natural compound derived from the marine sponge* Hyrtios* sp. Heteronemin triggers A498 cell cytotoxicity, growth inhibition, and apoptosis in a concentration- and time-dependent manner. Heteronemin-induced cell apoptosis is mediated by the release of cytochrome *c* into the cytosol upon the disruption of the mitochondria membrane potential. Cytosolic cytochrome *c* mediates the allosteric activation of Apaf-1, and together they form the apoptosome. The apoptosome recruits caspase-9 leading to the amplification of the apoptotic signal through the proteolytic cleavage and the activation of capase-9 and caspase-3 [40].

Bcl-2 family proteins are involved in the regulation of the outer mitochondrial membrane permeabilization and are divided into two groups: pro- and antiapoptotic proteins. Proapoptotic proteins, such as Bax and Bak, homooligomerize and participate in the formation of pores in the outer mitochondrial membrane through which proapoptotic molecules escape, including second mitochondria-derived activator of caspase (Smac) (also known as Diablo) and cytochrome *c*. Antiapoptotic proteins, such as Bcl-2 and Bcl-xL, block cell death by preventing the activation and homooligomerization of Bax and Bak [41]. We found that heteronemin downregulates the expression of Bcl-2 and Bcl-xL, upregulates the expression of Bax, and induces the loss of mitochondria membrane potential. These results suggest that the mitochondrial apoptotic pathway is important in heteronemin-induced cell death in A498 cells.

The PI3K/AKT signaling pathway regulates many normal cellular functions that are also critical for tumorigenesis, including cell proliferation, growth, survival, and motility. Several studies show that PI3K/AKT pathway is abnormally overexpressed in RCC [42–45], making it an attractive target for anticancer therapy [46–48]. For cell survival, AKT activates the NF-*κ*B pathway via the regulation of I*κ*B kinase (IKK), resulting in transcription of prosurvival genes. The transcription factor NF-*κ*B transactivates various antiapoptotic NF-*κ*B target genes, including inhibitor of apoptosis proteins (IAPs), Bcl-xL, and Bcl-2 [49]. A previous study had reported that heteronemin completely prevents TNF*α*-induced degradation of I*κ*B*α* and the subsequent translocation of p50 and p65 to the nucleus and that heteronemin is able to inhibit the activation of the NF-*κ*B pathway [10]. Our study shows that heteronemin treatment markedly suppresses the activation of AKT and its related downstream proteins. The ability of heteronemin to inhibit the AKT and NF-*κ*B pathways demonstrates that heteronemin is an effective bioactive marine natural compound.

The p38 signaling pathway is closely associated with the initiation of apoptosis in various types of cells, and this pathway is the target of many antitumor compounds [24]. AD-1, a novel ginsenoside derivative, increased the phosphorylation level of p38 which contributed to the antiproliferative effect, and* in vivo* data showed that treatment of AD-1 led to p38 activation, which correlated with decreased angiogenesis and the inhibition of tumor growth [50]. Arctigenin, a dietary phytoestrogen, increased superoxide anion and hydrogen peroxide levels by nicotinamide adenine dinucleotide phosphate (NADPH) oxidase 1 (NOX1) and activated p38 pathway to induce apoptosis in human breast cancer MDA-MB-231 cells by triggering the mitochondrial caspase-independent apoptotic pathway [51]. In addition to apoptosis, p38 can also mediate autophagy in response to chemotherapeutic agents. However, the treatment with p38 inhibitor had no influence on the expression of LC3 in our model (Figure S2). Here, we found that heteronemin rapidly activates p38 and the inhibition of p38 reverses heteronemin-induced cell apoptosis, demonstrating that p38 is involved in the cell apoptotic pathway but not in the autophagic pathway.

At low levels, autophagy is a process that allows cells to adapt to stress and avoid cell death; however, at high levels and under some cellular circumstances, autophagy offers an alternative pathway to kill abnormal cells. We observed autophagy induction in A498 cells after heteronemin treatment, as evidenced by the upregulation of LC3-II protein. However, the addition of the autophagy inhibitor chloroquine increased heteronemin cytotoxicity in A498 cells, suggesting a potential avenue for an enhanced therapeutic activity. It has been reported that autophagy inhibitors given in combination with chemotherapy suppressed tumor growth and triggered cell death to a greater extent than did chemotherapy alone, both* in vitro* and* in vivo* [52]. These data indicate that the prosurvival autophagy is a novel therapeutic target. Moreover, several studies have shown that the JNK pathway also induces autophagy. JNK signaling was a key requirement for upregulation of LC3 during ceramide-induced autophagy in human nasopharyngeal carcinoma cells [26]. Bortezomib induced autophagy in head and neck squamous cell carcinoma cells via JNK activation [53]. We found that heteronemin induces the phosphorylation of JNK and the cotreatment with a JNK inhibitor increases heteronemin-induced cytotoxicity and apoptotic signaling in A498 cells at a level similar to that with chloroquine. The activation of JNK modulates autophagy through two distinct mechanisms. (i) It promotes the phosphorylation of Bcl-2/Bcl-xL resulting in the dissociation of the Beclin 1-Bcl-2/Bcl-xL complex, thereby stimulating autophagy [54]. (ii) JNK leads to the upregulation of damage-regulated autophagy modulator (DRAM). DRAM can stimulate the accumulation of autophagosomes by regulating the autophagosome-lysosome fusion to generate autolysosomes [55]. Therefore, the crosstalk between JNK activation and heteronemin-induced autophagy needs to be further investigated.

**Figure 8:**
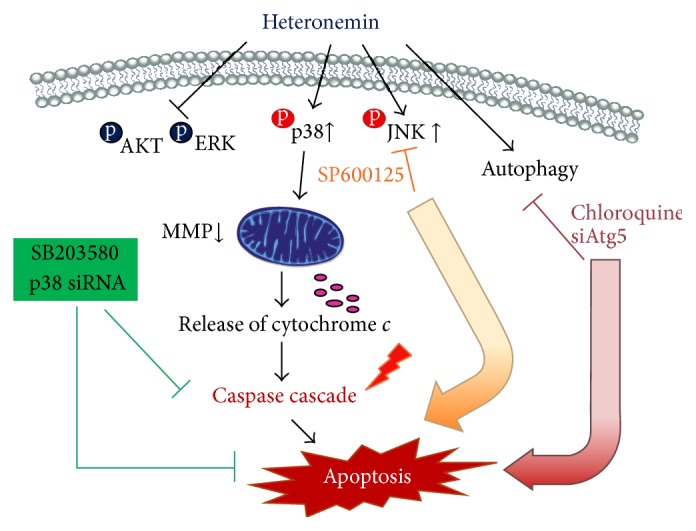
Schematic representation of the different pathways shown in this report to be activated by heteronemin leading to apoptosis in A498 cells.

Taken together, this study shows that heteronemin induces apoptosis and autophagy in human renal carcinoma A498 cells. Heteronemin inhibits the phosphorylation of ERK and AKT signaling pathway and increases the phosphorylation of p38 and JNK. The inhibition of p38, but not JNK, can reverse heteronemin-induced cytotoxicity and apoptotic signaling. Heteronemin also induces autophagy in A498 cells, and cotreatment with chloroquine or SP600125 inhibits autophagy and increases heteronemin-induced cytotoxicity and apoptotic signaling (Figure 8). Therefore, this investigation provides new insight into the role of heteronemin as a potential anticancer agent and suggests that the combination of heteronemin with autophagy inhibitors further enhances its therapeutic effects.

## Supplementary Material

Supplementary Figure S1: Effects of heteronemin on cell cycle distribution in A498 cells. Cells were incubated with (A) DMSO or various concentrations of heteronemin for 24 h and (B) DMSO or 3 μM heteronemin for the indicated time periods. Cell cycle phase and cell apoptosis were determined by FACS as described in Materials and Methods.
Supplementary Figure S2: Effects of SB203580 on heteronemin-induced LC3 conversion in A498 cells. Cells were incubated with DMSO, heteronemin 3 μM, SB203580 25 μM and combination of heteronemin and SB203580 for 24 h and detected protein expression by western blotting. DMSO was used as the vehicle control (CTL).
