# Supplementary material for: Heteronemin, a Spongean Sesterterpene, Induces Cell Apoptosis and Autophagy in Human Renal Carcinoma Cells
Source: Biomed Res Int. 2015 May 18;2015:738241. doi: 10.1155/2015/738241 (PMC4450260; doi:10.1155/2015/738241)
Supplement: Supplementary file 1 — Supplementary Figure S1: Effects of heteronemin on cell cycle distribution in A498 cells. Cells were incubated with (A) DMSO or various concentrations of heteronemin for 24 h and (B) DMSO or 3 μM heteronemin for the indicated time periods. Cell cycle phase and cell apoptosis were determined by FACS as described in Materials and Methods. Supplementary Figure S2: Effects of SB203580 on heteronemin-induced LC3 conversion in A498 cells. Cells were incubated with DMSO, heteronemin 3 μM, SB203580 25 μM and combination of heteronemin and SB203580 for 24 h and detected protein expression by western blotting. DMSO was used as the vehicle control (CTL). [file 738241.f1.pdf]

Figure S1

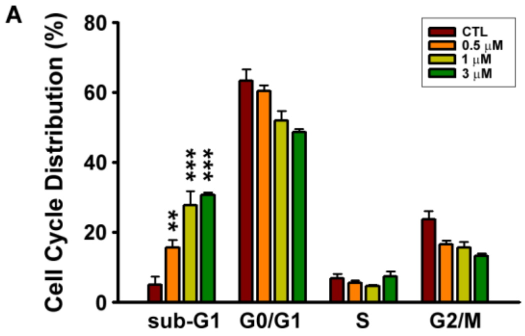

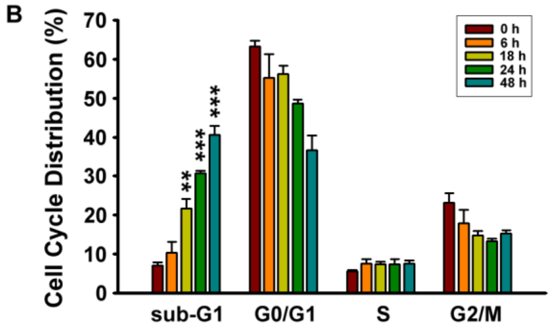

Fig S1. Effects of heteronemin on cell cycle distribution in A498 cells. Cells were incubated with vehicle or variou concentrations of heteronemin for 24 h or indicated time and detected cell cycle distribution by flow cytometry.

Figure S2

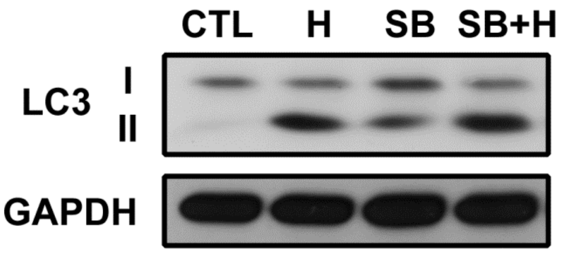

Fig S2. Effects of SB203580 on heteronemin-induced LC3 conversion in A498 cells. Cells were incubated with vehicle, heteronemin 3 μM, SB203580 25 μM and combination of heteronemin and SB203580 for 24 h and detected protein expression by western blotting.
